# The overlooked burden: Vaginismus and its greater prevalence in eastern women

**DOI:** 10.1016/j.dialog.2025.100205

**Published:** 2025-01-17

**Authors:** Hafsa Nasim, Abdulqadir J. Nashwan

**Affiliations:** a Department of Medicine, Services Institute of Medical Sciences, Lahore 54000, Pakistan; b Department of Nursing, Hamad Medical Corporation, Doha, Qatar

**Keywords:** Vaginismus, Female sexual dysfunction, Eastern countries

To the Editor,

Vaginismus is a female sexual dysfunction (FSD) condition with a clinical prevalence rate of 5–7 % worldwide but a comparatively higher prevalence in Eastern countries [1,2]. Vaginismus is a multi-dimensional disorder in which there are reflex or involuntary muscle contractions of the pelvic floor muscles, especially those in the outer one-third of the vagina, caused by attempts at vaginal penetration [3,4]. It mostly affects younger women (ages 20–35) and women who are new to vaginal intercourse and in women aged above 45 (postmenopausal women) [5,6]. This condition may lead to problems or pain during sexual activity, tampon insertion, or gynecologic exams [7]. Most patients suffering from this condition experience extreme fear of having sex or have psychological symptoms like low self-esteem, embarrassment, hesitation, and negative symptoms [1,8]. Vaginismus is a phobia, as it is a persistent, excessive, or unreasonable fear triggered by the presence or anticipation of intercourse [9]. It is among the most significant conditions that have a negative effect on a woman's and her partner's lifestyle, reproductive health, and mental health, as it leads to sterility and unconsummated marriage [7,9].

The prevalence of vaginismus varies between 5 % and 17 % in the United States. Eastern nations, on the other hand, claim much greater rates: 20 % in Egypt, 27 % in Iran, 43 % in Turkey, and 68 % in Ghana [2,5,8,10]. Vaginismus prevalence is often underestimated due to challenges in data collection, clinical surveys, and cultural stigma, which hinder accurate assessments due to feelings of guilt and secrecy. Studies, including those by McEvoy et al., reveal that conservative moral standards and beliefs about sexuality significantly influence the condition, with many patients indicating that upbringing contributes to the perception of sex as wrong, alongside pain-related fears. Such societal factors may shape women's experiences with vaginismus, impacting their management and understanding of the condition [2]. Management often involves multimodal techniques such as education, pharmacological therapy, cognitive behavioral therapy like Senate Focus (a set of structured touching exercises meant to help couples overcome their nervousness and become more comfortable with physical intimacy), physical therapy, surgery, and sometimes administration of Botox injection [9,11,12]. Vaginismus, despite its widespread prevalence, is still poorly understood [2].

The cultural barrier and lack of awareness contribute largely to the fact that vaginismus is more rampant in women from Eastern countries. Sex education concerning all extents of sexual activities similar to the Western countries can help in the elimination of stigma, fostering common understanding, and empowering the women in society for early diagnosis and proper treatment. For example a study by Banaei et al. proved that competent sex education enhances adolescents' attitude and can solve sexual issues by providing a foundation for optimum sexual health clinics and treatment plan [13]. An other example is findings of Pithavadian's publication according to which during medical consultations, raise awareness of vaginismus, debunk sex-related beliefs, de-stigmatize vaginismus, and empower those who have it. These were found to be practical methods that participants suggested to enhance vaginismus health care and help-seeking [14].The utilization of media or high-level social campaigns for the eradication of sexual health-related stigma should also be made effective. Reproductive-age women should be the target of educational campaign events. It is still not well characterized, and more research is needed, especially in postmenopausal women.

## CRediT authorship contribution statement

**Hafsa Nasim:** Writing – review & editing, Writing – original draft. **Abdulqadir J. Nashwan:** Writing – review & editing, Writing – original draft.

## Declaration of competing interest

The authors declare that they have no known competing financial interests or personal relationships that could have appeared to influence the work reported in this paper.
